# Cardiometabolic multimorbidity, genetic risk, and dementia: a prospective cohort study

**DOI:** 10.1016/S2666-7568(22)00117-9

**Published:** 2022-06

**Authors:** Xin You Tai, Michele Veldsman, Donald M Lyall, Thomas J Littlejohns, Kenneth M Langa, Masud Husain, Janice Ranson, David J Llewellyn

**Affiliations:** a Nuffield Department of Clinical Neurosciences, University of Oxford, Oxford, UK; b Department of Experimental Psychology, University of Oxford, Oxford, UK; c Wellcome Centre for Integrative Neuroimaging, University of Oxford, Oxford, UK; d Nuffield Department of Population Health, University of Oxford, Oxford, UK; e Division of Clinical Neurology, John Radcliffe Hospital, Oxford University Hospitals Trust, Oxford, UK; f Institute of Health and Wellbeing, University of Glasgow, Glasgow, UK; g Department of Internal Medicine, School of Medicine, University of Michigan, Ann Arbor, MI, USA; h Institute for Social Research, University of Michigan, Ann Arbor, MI, USA; i Institute for Healthcare Policy and Innovation, University of Michigan, Ann Arbor, MI, USA; j Veterans Affairs Ann Arbor Center for Clinical Management Research, Ann Arbor, MI, USA; k College of Medicine and Health, University of Exeter, Exeter, UK; l Alan Turing Institute, London, UK

## Abstract

**Background:**

Individual cardiometabolic disorders and genetic factors are associated with an increased dementia risk; however, the relationship between dementia and cardiometabolic multimorbidity is unclear. We investigated whether cardiometabolic multimorbidity increases the risk of dementia, regardless of genetic risk, and examined for associated brain structural changes.

**Methods:**

We examined health and genetic data from 203 038 UK Biobank participants of European ancestry, aged 60 years or older without dementia at baseline assessment (2006–10) and followed up until March 31, 2021, in England and Scotland and Feb 28, 2018, in Wales, as well as brain structural data in a nested imaging subsample of 12 236 participants. A cardiometabolic multimorbidity index comprising stroke, diabetes, and myocardial infarction (one point for each), and a polygenic risk score for dementia (with low, intermediate, and high risk groups) were calculated for each participant. The main outcome measures were incident all-cause dementia and brain structural metrics.

**Findings:**

The dementia risk associated with high cardiometabolic multimorbidity was three times greater than that associated with high genetic risk (hazard ratio [HR] 5·55, 95% CI 3·39–9·08, p<0·0001, and 1·68, 1·53–1·84, p<0·0001, respectively). Participants with both a high genetic risk and a cardiometabolic multimorbidity index of two or greater had an increased risk of developing dementia (HR 5·74, 95% CI 4·26–7·74, p<0·0001), compared with those with a low genetic risk and no cardiometabolic conditions. Crucially, we found no interaction between cardiometabolic multimorbidity and polygenic risk (p=0·18). Cardiometabolic multimorbidity was independently associated with more extensive, widespread brain structural changes including lower hippocampal volume (F_2, 12 110_ = 10·70; p<0·0001) and total grey matter volume (F_2, 12 236_ = 55·65; p<0·0001).

**Interpretation:**

Cardiometabolic multimorbidity was independently associated with the risk of dementia and extensive brain imaging differences to a greater extent than was genetic risk. Targeting cardiometabolic multimorbidity might help to reduce the risk of dementia, regardless of genetic risk.

**Funding:**

Wellcome Trust, Alzheimer's Research UK, Alan Turing Institute/Engineering and Physical Sciences Research Council, the National Institute for Health Research Applied Research Collaboration South West Peninsula, National Health and Medical Research Council, JP Moulton Foundation, and National Institute on Aging/National Institutes of Health.

## Introduction

Stroke, diabetes, and myocardial infarction are strong independent cardiometabolic risk factors for dementia.^1^, ^2^, ^3^, ^4^, ^5^, ^6^, ^7^ Cardiometabolic multimorbidity, defined as two or more cardiometabolic conditions, is rapidly rising in prevalence^8^, ^9^ and is strongly predictive of mortality.^10^ However, the extent to which cardiometabolic multimorbidity predicts the risk of dementia and associated brain structural changes is largely unknown. Additionally, it is unclear how the strength of this association compares or interacts with the genetic risk of dementia.

Most adult dementia cases are sporadic, wherein no single gene mutation is considered causative, and multiple genes influence risk.^11^ A meta-analysis of genome-wide association studies (GWAS) of people of European ancestry identified risk loci associated with late-onset Alzheimer's disease,^12^ in addition to the widely known ε4 allele of the apolipoprotein E (*APOE*) gene.^13^ Polygenic risk scores provide a useful quantification of genetic risk by combining multiple risk alleles of generally small but cumulative effect and have shown to be predictive for all-cause dementia.^14^ Although some studies examining polygenic risk and upstream lifestyle or cardiometabolic risk factors suggest no interaction between genes and environment,^15^, ^16^ one study found the opposite.^17^ To our knowledge, no previous study has examined the relationship between cardiometabolic multimorbidity, polygenic risk, and dementia incidence.

In this study, we analysed data from the UK Biobank prospective cohort to test the hypothesis that both cardiometabolic multimorbidity and genetic risk were independently associated with the risk of incident dementia and brain structural measures. We investigated the extent to which low cardiometabolic multimorbidity is associated with a reduced risk of dementia among participants with different genetic risk, as this could have important implications for a targeted approach for dementia risk reduction, considering an individual's genetic risk.

**Research in context**

**Evidence before this study**

We searched PubMed, Web of Science, and Google Scholar for research published in English from inception to Dec 12, 2021, using the terms “(dementia OR alzheimer's disease) AND (cardiovascular OR metabolic OR cardiometabolic) AND (multimorbidity)”. Observational studies identified individual cardiovascular or metabolic conditions, such as myocardial infarction, as independent risk factors for dementia. There is a large amount of literature examining upstream cardiovascular risk factors, such as high blood pressure, and lifestyle factors on dementia risk. However, for various reasons, including small sample sizes, the relationship between multiple cardiometabolic conditions and the risk of developing dementia is largely unknown. This paucity of research is important in the context of rising prevalence of both dementia and cardiometabolic multimorbidity, especially in low-income and middle-income countries. Furthermore, it is unclear how this relationship compares or interacts with the genetic risk of dementia, with mixed findings when examining upstream cardiovascular risk factors and dementia risk.

**Added value of this study**

With the largest sample to date known to have addressed this issue, this study extends existing knowledge by examining the association between multiple established cardiometabolic conditions and the risk of developing dementia, as well as the potential interaction with genetic risk in individuals aged 60 years and older. The risk associated with developing dementia in participants with cardiometabolic multimorbidity of stroke, myocardial infarction, and diabetes was more than three times higher than the risk of developing dementia in participants with a high genetic risk. We found no interaction between cardiometabolic multimorbidity and genetic risk for both dementia risk and structural brain health.

**Implications of all the available evidence**

Our results suggest the importance of targeting cardiometabolic multimorbidity in reducing dementia risk, regardless of predetermined genetic risk. These findings have important implications for clinical practice and public health initiatives in dementia prevention and care.

## Methods

### Study design and population

This cohort study is based on data from the UK Biobank,^18^ which received approval from the National Information Governance Board for Health and Social Care and the National Health Service North West Multicentre Research Ethics Committee. All participants provided written informed consent through electronic signature.

The UK Biobank is a population-based cohort of more than 500 000 participants aged 40–73 years, who underwent assessment at one of 22 centres across the UK between 2006 and 2010.^18^ Participants provided biological samples, completed touch-screen questionnaires, and underwent physical examination. A subgroup of participants re-attended for brain imaging between 2014 and 2020.^19^ For this study, analyses were restricted to individuals aged at least 60 years at baseline who had genetic information available (n=203 038, and an imaging subsample of 12 236 participants). We restricted the analysis to participants aged 60 years or older as most sporadic dementia occurs in older individuals, and to reduce the likelihood of identifying cases with a monogenic risk of early-onset dementia. Participants with prevalent dementia at baseline or a history of CNS infection, encephalitis, meningitis, amyotrophic lateral sclerosis, multiple sclerosis, or previous subdural or subarachnoid haemorrhage were excluded from the study. All baseline diagnoses in this study were based on a combination of self-report and hospital inpatient records (information on UK Biobank codes are found in the appendix p 2). Certain variables, such as stroke diagnosis, were previously derived by the UK Biobank by combining these different sources of information and have been validated elsewhere.^20^

### Cardiometabolic multimorbidity

Our primary objective was to investigate the relationship between stroke, diabetes, and myocardial infarction and dementia risk, as several studies have shown that the combination of these conditions contributes substantially to mortality,^10^ and separately to dementia risk.^1^, ^2^ First, to explore the effect of each condition, we derived an eight-level categorical variable by separating participants into mutually exclusive groups according to baseline prevalent diseases of stroke; myocardial infarction; diabetes; stroke and myocardial infarction; stroke and diabetes; myocardial infarction and diabetes; diabetes, stroke, and myocardial infarction; or none of these (the reference group). Next, a cardiometabolic multimorbidity index was calculated for each participant, whereby the three cardiometabolic conditions were considered as separate binary variables and a point value was assigned based on the presence or absence of the condition, ranging from 0 (none of the conditions) to 3 (all conditions). This approach allowed better comparison with polygenic risk.

### Dementia diagnosis

All-cause dementia was established using hospital inpatient records or from death register linkage data as an underlying or contributory cause. Diagnoses were documented using the International Classification of Diseases (ICD) coding system, using ICD-9 and ICD-10 codes for Alzheimer's disease and other dementia classifications (appendix p 2).

### Polygenic risk score

A polygenic risk score, reflecting each participant's Alzheimer's disease-related genetic risk, was constructed as has been described in detail previously.^15^ The polygenic risk score was based on the results of a GWAS of individuals of European ancestry and includes the *APOE* region.^12^ Therefore, the present study was restricted to individuals whose self-reported racial or ethnic background was White (British, Irish, or other White background). Single nucleotide polymorphisms (SNPs) associated with Alzheimer's disease that were common and available in the UK Biobank were selected using so-called clumped results, referring to the most significant variant per linkage disequilibrium block (n=249 273, with an inclusion threshold p<0·5). The number of associated alleles were weighted according to their association strength with Alzheimer's disease,^12^ summed, and then Z-standardised to derive a polygenic risk score. Polygenic risk was grouped according to quintiles as low risk (quintile 1), intermediate risk (quintiles 2, 3, and 4), and high risk (quintile 5).

### Covariates

All models were adjusted for age (continuous), sex (female *vs* male), education (categorised as higher [college or university degree or other professional qualification], upper secondary [second or final stage of secondary education], lower secondary [first stage of secondary education], vocational [work-related qualifications], or other), socioeconomic status (categories derived from Townsend deprivation index^21^ quintiles 1, 2 to 4, and 5, combining information on social class, employment, car availability, and housing), third-degree relatedness of individuals in the sample, the first 20 principal components of ancestry, and assessment centre. Models, including the polygenic risk score, were adjusted for the number of alleles included in the score to account for SNP-level variation.^15^

### Brain imaging measures

MRI data were acquired on a Skyra 3T scanner (Siemens; Munich, Germany) including high-resolution, T1-weighted, three-dimensional magnetisation-prepared gradient echo structural images and T2-weighted fluid-attenuated inversion recovery images. The full imaging protocol and processing pipeline have been previously described.^22^ We used imaging summary statistics (imaging-derived phenotypes) of total hippocampal volume, total grey matter volume, and white matter hyperintensity. Median absolute deviation was used to exclude outliers,^23^ and volumes were adjusted for potentially confounding baseline measures of age, age squared, head size, and imaging site.^22^

### Statistical analysis

Hazard ratios (HRs) were calculated using Cox proportional hazards regression models with time to incident all-cause dementia as the dependent variable. We calculated HRs for each of the seven mutually exclusive cardiometabolic multimorbidity groups compared with the baseline group with no cardiometabolic conditions. For our main model, we tested the association between the cardiometabolic multimorbidity index and genetic risk groups and time to incident all-cause dementia (nine categories with low genetic risk and cardiometabolic multimorbidity index 0 as reference). Cardiometabolic multimorbidity indices of two and three were combined because of small sample sizes. The proportionality of hazards assumption was assessed using the Schoenfeld residuals technique^24^ and satisfied (p=0·29 for testing departures from proportionality). Complete case analysis was applied to missing or not known data for all exposures and covariates with less than 3% missing values. For categorical variables, (education and the Townsend deprivation index) we included missing as a separate category. Participants were considered at risk for dementia from baseline until the date of first diagnosis, death, loss to follow-up, or the last surveyed hospital admission date (March 31, 2021, for England and Scotland and Feb 28, 2018, for Wales), whichever came first. These censoring dates were recommended by UK Biobank for when the data was estimated to be over 90% complete in England, Scotland, and Wales. Information was accurate up to these dates and did not rely on multiple research reassessments.

Total hippocampal and grey matter volume was normally distributed, and total white matter hyperintensity volume was log-transformed to correct for a positively skewed distribution. Two-way ANOVA was done to examine the association for cardiometabolic multimorbidity index (0, 1, or ≥2) and polygenic risk (low, intermediate, or high) as predictors with each brain measure as the outcome.

Secondary data analyses examined how additional cardiovascular conditions of atrial fibrillation, heart failure, and peripheral vascular disease would change the association of the three main cardiometabolic conditions and dementia risk. We also considered myocardial infarction, atrial fibrillation, and heart failure as a single composite heart disease variable in a separate sensitivity analysis. One model included additional covariates of systolic blood pressure, total cholesterol level, body-mass index (BMI), and glycosylated haemoglobin (HbA_1c_) to examine the extent to which these upstream cardiometabolic risk markers might explain the associations between cardiometabolic multimorbidity and dementia risk. Further sensitivity analyses for the risk of incident dementia used genetic risk quintiles,^15^ to check for differences with grouping genetic risk into three groups, and genetic risk adjusting for *APOE* ε4 allele status. Analyses stratified by different follow-up durations of up to 10 years and 10–14 years was done to consider an earlier risk of developing dementia and potential reverse causation, respectively.

A post-hoc analysis investigated pairwise differences between individual levels of cardiometabolic multimorbidity and polygenic risk using Tukey and Holm–Bonferroni correction for pairwise and multiple comparisons. A linear regression model was used to control for all remaining baseline covariates. p values were two-sided with statistical significance set at p<0·05. Analyses were done in Matlab R2018a or in R version 4.0.3 using the survival package.

### Role of the funding source

The funders of this study had no role in study design, data collection, data analysis, data interpretation, or writing of the report.

## Results

The UK Biobank baseline sample comprised 502 536 participants. After excluding participants younger than 60 years (274 748 participants), those with prevalent dementia at baseline (103 participants), those with no genetic data (6630 participants), those of non-European descent (15 367 participants), or those who fulfilled any other exclusion criteria (2650 participants), 203 038 participants were included in our study sample (appendix p 4). The mean patient age was 64·9 years (SD 3·0) and 107 243 (52·8%) of 203 038 participants were female (table). Over 2 354 857 follow-up person-years (median 12·0, IQR 11·2–12·7), 4766 cases of all-cause incident dementia were observed. The polygenic risk score was normally distributed across participants and was not associated with any cardiometabolic condition (appendix p 5).

**Table:** Baseline characteristics

		**No incident dementia (n=198 272)**	**Incident dementia (n=4766)**
Age, years		64·9 (3·0)	66·6 (2·8)
Sex			
	Female	105 017 (53·0%)	2226 (46·7%)
	Male	93 255 (47·0%)	2540 (53·3%)
Education*			
	Higher	64 750 (32·7%)	1198 (25·1%)
	Upper secondary	14 361 (7·2%)	352 (7·4%)
	Lower secondary	17 880 (9·0%)	397 (8·3%)
	Vocational	46 240 (23·3%)	929 (19·5%)
	Other	55 041 (27·8)	1890 (39·7)
Socioeconomic status quintile†			
	1 (least deprived)	39 731 (20·0%)	833 (17·5%)
	2–4	119 043 (60·0%)	2689 (56·4%)
	5 (most deprived)	39 335 (19·8%)	1239 (26·0%)
	Other	163 (0·1%)	5 (0·1%)
Cardiometabolic conditions			
	Stroke only	3006 (1·5%)	157 (3·3%)
	Diabetes only	11 068 (5·6%)	524 (11·0%)
	Myocardial infarction only	5646 (2·8%)	235 (4·9%)
	Stroke and myocardial infarction	508 (0·3%)	50 (1·0%)
	Stroke and diabetes	598 (0·3%)	66 (1·4%)
	Myocardial infarction and diabetes	1372 (0·7%)	107 (2·2%)
	Stroke and myocardial infarction and diabetes	122 (0·1%)	16 (0·3%)
	No cardiometabolic conditions	176 318 (88·9%)	3659 (76·8%)
Number of cardiometabolic conditions‡ (cardiometabolic multimorbidity index)			
	0	176 318 (88·9%)	3659 (76·8%)
	1	19 720 (9·9%)	916 (19·2%)
	2	2112 (1·1%)	175 (3·7%)
	3	122 (0·1%)	16 (0·3%)
Genetic risk category§			
	Low	39 879 (20·1%)	729 (15·3%)
	Intermediate	119 019 (60·0%)	2803 (58·8%)
	High	39 374 (19·9%)	1234 (25·9%)

Data are mean (SD) or n (%). Percentages might not sum to 100 because of rounding.

* Higher education is defined as college or university degree or other professional qualification; upper secondary as the second or final stage of secondary education; lower secondary as the first stage of secondary education; and vocational as work-related practical qualifications.

† Socioeconomic status assessed with the Townsend deprivation index,^21^ which combines information on social class, employment, car availability, and housing.

‡ Cardiometabolic multimorbidity index groups are mutually exclusive.

§ Genetic risk categories were defined according to a polygenic risk score as low (lowest quintile), intermediate (quintiles 2 to 4), or high (highest quintile).

At baseline assessment, 3163 (1·6%) of 203 038 participants had a history of stroke only, 11 592 (5·7%) had a history of diabetes only, 5881 (2·9%) had a history of myocardial infarction only, 420 (0·2%) had both stroke and myocardial infarction, 526 (0·3%) had both stroke and diabetes, 1341 (0·7%) had both myocardial infarction and diabetes, and 138 (0·1%) had diabetes, stroke, and myocardial infarction.

**Figure 1:**
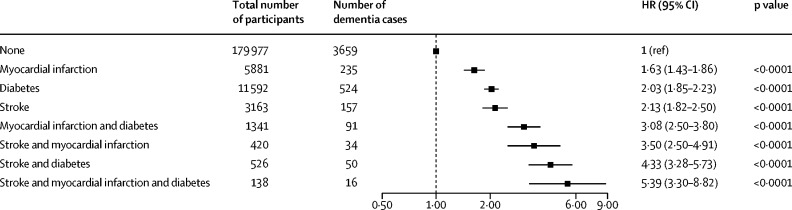
Risk of incident dementia by cardiometabolic disease status at baseline Data are HRs for incident dementia, associated with mutually exclusive groupings of cardiometabolic conditions. The model has been adjusted for age, sex, education, socioeconomic status, relatedness, number of alleles included in the polygenic risk score, first 20 principal components of ancestry, and assessment centre. Unadjusted model results are shown in the appendix (p 13). HR=hazard ratio.

Compared with the reference group with no cardiometabolic conditions, the adjusted HRs for dementia incidence were 2·13 (95% CI 1·82–2·50) for participants with a history of stroke only, 2·03 (1·85–2·23) for those with diabetes only, and 1·63 (1·43–1·86) for those with myocardial infarction only. For participants with multimorbidity, the adjusted HRs for dementia incidence were 3·50 (95% CI 2·50–4·91) in those with stroke and myocardial infarction, 4·33 (3·28–5·73) in those with stroke and diabetes, 3·08 (2·50–3·80) in those with both myocardial infarction and diabetes, and 5·39 (3·30–8·82) in those with stroke, diabetes, and myocardial infarction (figure 1).

We assigned a cardiometabolic multimorbidity index based on the presence of each cardiometabolic condition. 20 636 (10·2%) of 203 038 participants had a cardiometabolic multimorbidity index of one, 2287 (1·1%) had a cardiometabolic multimorbidity index of two, and 138 (0·1%) had a cardiometabolic multimorbidity index of three (table 1).

The risk of developing dementia increased monotonically with increasing cardiometabolic multimorbidity index. Compared with baseline, participants with a cardiometabolic multimorbidity index of one had an adjusted HR for dementia incidence of 1·94 (95% CI 1·80–2·08, p<0·0001), participants with a cardiometabolic multimorbidity index of two had an adjusted HR of 3·46 (2·97–4·04, p<0·0001), and participants with a cardiometabolic multimorbidity index of three had an adjusted HR of 5·55 (3·39–9·08, p<0·0001). In terms of genetic risk, the adjusted HR for dementia incidence was 1·27 (95% CI 1·17–1·38, p<0·0001) for participants with intermediate polygenic risk and 1·68 (1·53–1·84, p<0·0001) for participants with high polygenic risk, compared with low genetic risk (appendix p 6).

**Figure 2:**
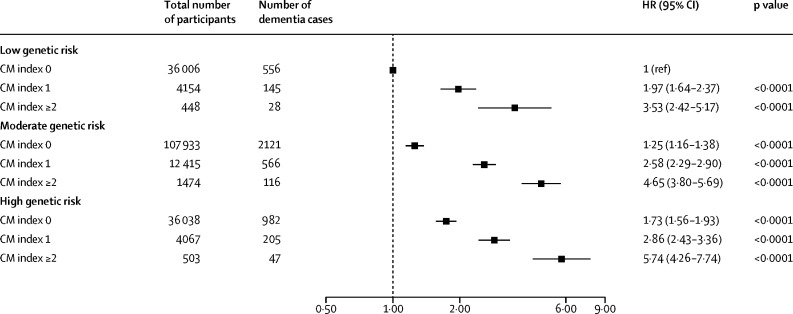
Risk of incident dementia according to CM index and genetic risk Data are HRs for incident dementia, according to CM index and genetic risk. The reference group was participants with low genetic risk and a CM index of 0. The model has been adjusted for age, sex, education, socioeconomic status, relatedness, number of alleles included in the polygenic risk score, first 20 principal components of ancestry, and assessment centre. Unadjusted model results are shown in the appendix (p 14). CM=cardiometabolic multimorbidity. HR=hazard ratio.

Within each genetic risk group, increasing cardiometabolic multimorbidity led to an increased risk of dementia (figure 2). 47 (9·3%) of 503 participants with high genetic risk and a cardiometabolic multimorbidity index of two or more developed dementia compared with 556 (1·5%) of 36 006 participants with low genetic risk and a cardiometabolic multimorbidity index of zero (HR 5·74, 95% CI 4·26–7·74). We found no significant interaction between cardiometabolic multimorbidity and polygenic risk (p=0·18), indicating that the association between cardiometabolic multimorbidity and dementia incidence was not substantially modified by genetic risk.

Similar patterns of association were observed in sensitivity analyses, including when considering the additional cardiovascular conditions of atrial fibrillation and heart failure (appendix p 7) or combining myocardial infarction, atrial fibrillation, and heart failure together as a composite heart disease variable (appendix p 8). Considering polygenic risk quintiles in the model (appendix p 9) and additionally adjusting for *APOE* ε4 allele status (appendix p 10) yielded similar associations with dementia risk. Stratified analyses based on follow-up duration showed a similar pattern in participants developing all-cause dementia within 10 years of follow-up and those developing dementia 10–14 years after baseline assessments (appendix p 12). The main difference was a non-significant relationship between individuals with stroke, myocardial infarction, and diabetes and the risk of dementia over 10–14 years of follow-up, probably due to the presence of only one incident dementia case in this subgroup. HRs were attenuated slightly after adjustment for markers of upstream cardiometabolic risk, including systolic blood pressure, total cholesterol level, BMI, and HbA_1c_ (appendix p 11), but the overall pattern of association was unchanged.

**Figure 3:**
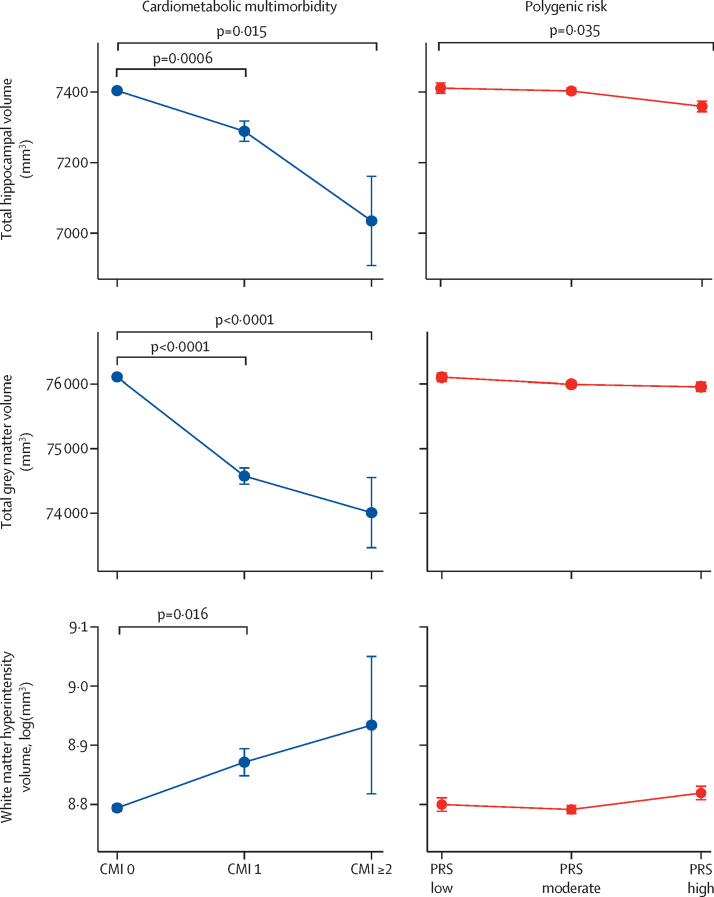
Total hippocampal volume, total grey matter volume, and white matter hyperintensity volume associated with CMI and polygenic risk Data are brain volume of the hippocampi, total grey matter, and white matter hyperintensities stratified according to CMI and polygenic risk. Error bars represent SEs. White matter hyperintensity volume has been log-transformed because of skewed distribution. CMI=cardiometabolic multimorbidity index*.* PRS=polygenic risk score.

We examined the relationship between cardiometabolic multimorbidity and polygenic risk and total hippocampal volume, total grey matter volume, and white matter hyperintensity volume, which represent regions of interest related specifically to Alzheimer's disease, whole brain structural integrity, and cerebrovascular burden, respectively (figure 3). Cardiometabolic multimorbidity was significantly associated with lower hippocampal volume (F_2, 12 110_=10·70; p<0·0001), total brain grey matter volume (F_2, 12 236_=55·65; p<0·0001), and higher white matter hyperintensity volume (F_2, 10 827_=4·48; p=0·011). By contrast, higher genetic risk was significantly associated with a lower hippocampal volume only (F_2, 12 110_=3·45; p=0·032), but not total grey matter (F_2, 12 236_=1·67; p=0·19) or white matter hyperintensity volume (F_2, 10 827_=0·98; p=0·38). No interaction was identified between cardiometabolic multimorbidity and polygenic risk for any of the brain measures (appendix p 15) and post-hoc analysis showed differences between individual indices of cardiometabolic multimorbidity and polygenic risk groups. A fully adjusted linear regression model accounting for all baseline characteristics showed the same pattern of associations (appendix p 16).

## Discussion

This large, population-based study adds to the existing knowledge base by examining how cardiometabolic multimorbidity and genetic risk are associated with dementia incidence. Cardiometabolic multimorbidity and genetic risk were independently associated with all-cause dementia. Therefore, within any genetic risk category, increasing cardiometabolic morbidity was associated with an additive risk of developing dementia. Cardiometabolic multimorbidity was also associated with lower total hippocampal and grey matter volumes, and higher white matter hyperintensity volume, whereas polygenic risk was associated with lower hippocampal volume only. As reported in the 2020 *Lancet* Commission on dementia prevention and care,^25^ there is a high prevalence of cardiovascular multimorbidity in patients with dementia. However, few studies have examined the association between multiple cardiometabolic conditions and dementia risk, with those that have being restricted by small sample size. Grande and colleagues^26^ identified an HR of 1·61 (95% CI 1·17–2·29) for developing dementia with multiple cardiovascular conditions,^26^ whereas another study reported an HR of 50·30 (14·57–173·57) with stroke and congestive cardiac failure^27^ in cohorts of 2478 individuals and 1701 individuals, respectively. Most studies have focused on upstream lifestyle or cardiometabolic risk factors,^28^, ^29^, ^30^ such as high blood pressure and blood glucose, and have shown that poor control of individual risk factors might be associated with up to a two times increased risk of developing dementia. A meta-analysis identified similar associations with dementia risk, including a pooled relative risk of 2·21 (95% CI 1·78 to 2·73) for three or more cardiovascular risk factors compared with none.^31^ In terms of lifestyle factors, in the same UK Biobank cohort as the present study, participants with an unhealthy lifestyle profile showed an increased HR of 1·35 (95% CI 1·15–1·58) compared with participants with a favourable risk profile, reflecting a significant but modest effect on dementia risk.^15^

Examining established cardiometabolic conditions provides a more complete understanding of the effect of upstream risk factors. In the present study, participants with only one cardiometabolic condition showed an HR for dementia risk of around 2, which is similar to previous reports examining diabetes,^1^ myocardial infarction,^2^ and stroke^3^ individually. Importantly, we observed that participants with all three conditions had more than a five times increase in all-cause dementia risk, suggesting a monotonic, additive relationship between increasing cardiometabolic multimorbidity and dementia risk, which was largely unchanged when considering upstream cardiometabolic risk factors. Therefore, the association between cardiometabolic multimorbidity and dementia risk is greater than that of combined lifestyle and cardiovascular risk factors and emphasises the importance of both clinical guidance and public health initiatives to aggressively prevent cardiometabolic disease, particularly in individuals with a pre-existing cardiometabolic condition.

To our knowledge, no previous study has examined the relationship between cardiometabolic multimorbidity, polygenic risk, and dementia incidence. Several studies have examined *APOE* ε4 status and individual cardiovascular conditions^27^ or risk factors,^32^ and one study investigated cardiovascular risk factors and a polygenic risk score based on 23 SNPs.^16^ Rasmussen and colleagues examined a comprehensive list of cardiovascular risk factors and genetic risk in relation to dementia and showed the highest 10-year absolute risk of all-cause dementia in individuals who smoked with diabetes, low education, *APOE* ε4, and 22–31 GWAS risk alleles.^33^ Results have been inconsistent because of restricted statistical power, as some studies indicated no interaction between genetic risk and cardiovascular risk factors^16^, ^33^ whereas others suggest factors such as midlife smoking increase dementia risk in *APOE* ε4 carriers but not non-carriers,^34^ and a favourable risk profile corresponded to a reduced dementia risk in *APOE* ε4 non-carriers only.^17^ Compared with previous work, the present study is an order of magnitude larger in sample size and incorporates a more comprehensive indicator of genetic risk. Our finding that the association of cardiometabolic multimorbidity with dementia incidence was both greater than, and independent of, genetic risk highlights the key message that risk factor modification appears critical for every individual, regardless of pre-determined genetic factors.

Previous studies have shown that individual cardiometabolic conditions, including stroke^35^ and diabetes,^36^ are associated with smaller overall brain volume, whereas high polygenic risk has been associated with smaller hippocampal volume in some cross-sectional studies^37^, ^38^ but not others.^39^ Our findings show that cardiometabolic multimorbidity was associated with larger and more widespread negative effects on brain measures, independent of differences associated with genetic risk. These findings, in combination with reports examining the contribution of vascular and neurodegenerative pathology on cognitive trajectories,^40^, ^41^ suggest that different mechanisms might underpin dementia risk. Cardiometabolic multimorbidity might result in combined global cerebrovascular and neurodegenerative processes,^42^ whereas genetic risk is more closely related to pathways involving pathological protein tau and β-amyloid aggregation, which have hippocampal specificity, especially during early stages of Alzheimer's dementia.^43^ More targeted studies are needed to fully elucidate this relationship but, nonetheless, the association of widespread, independent brain changes provides further reasons to address cardiometabolic multimorbidity.

This study should be considered in the context of several limitations. Because of the observational nature of our study, the association between cardiometabolic multimorbidity and dementia risk cannot be taken as causal. The UK Biobank cohort is more likely to be from less deprived areas and have fewer health conditions, such as cardiovascular disease, than the general population.^44^ The effects of cardiovascular risk and incident cardiovascular disease might potentially be larger in more representative cohorts because of this selection bias. However, there might also be a survivor bias, in that, if the incident cardiovascular disease is very severe, individuals might die before developing dementia. Although analyses were adjusted for various known potential confounders and participants were followed up for a median of 12 years, there remains a possibility of unmeasured confounding and reverse causation. Medical condition information was obtained from self-report or from medical records, where there was risk of incorrect reporting or missed diagnoses.^45^ The future release of comprehensive primary care data in the UK Biobank might help corroborate medical condition information. We examined polygenic risk derived for Alzheimer's disease and this has previously been shown to be predictive for all-cause dementia.^14^ The relevance of this polygenic risk score with brain regions related to Alzheimer's disease is less certain; however, research has shown corroborative hippocampal findings^37^ and suggests this is an appropriate comparison. Examining the relationship between cardiometabolic multimorbidity and dementia subtypes would be interesting; however, subtypes are currently poorly captured by medical records and dementia cases are likely to be Alzheimer's disease, vascular, or a mixed picture.^20^ Although this is one of the largest samples to investigate this question with a considerable follow-up period, certain study groups have low numbers, which might lead to a potential bias. and studies with larger subgroups would be useful to corroborate our results. Our study was restricted to participants of European ancestry, aged 60–73 years at baseline, and further research is needed to investigate whether these findings generalise to other populations. Neuroimaging analyses were cross-sectional and further research is necessary to establish the prospective association between cardiometabolic multimorbidity and dementia-related imaging features.

In conclusion, both cardiometabolic multimorbidity and genetic risk were significantly and independently associated with an increased risk of dementia and associated neuroimaging features. Future interventions targeting cardiometabolic risk factors might in turn prove effective in preventing dementia, regardless of genetic predisposition.

## Data sharing

UK Biobank data are available to all researchers for health-related research and public interest. Data can be accessed through the Access Management System (details at https://www.ukbiobank.ac.uk/enable-your-research/register). The variables used are detailed in the appendix (p 2). This research was done using the UK Biobank resource under application 9462.

## Declaration of interests

We declare no competing interests.
