# Supplementary material for: Cardiometabolic multimorbidity, genetic risk, and dementia: a prospective cohort study
Source: Lancet Healthy Longev. 2022 Jun;3(6):e428–36. doi: 10.1016/S2666-7568(22)00117-9 (PMC9184258; doi:10.1016/S2666-7568(22)00117-9)
Supplement: Supplementary appendix [file mmc1.pdf]

THE LANCET
Healthy Longevity

Supplementary appendix

This appendix formed part of the original submission and has been peer reviewed. We post it as supplied by the authors.

Supplement to: Tai XY, Veldsman M, Lyall DM, et al. Cardiometabolic multimorbidity, genetic risk, and dementia: a prospective cohort study. *Lancet Healthy Longev* 2022; **3:** e428–36.

Supplementary material

Table S1. Codes used in the UK Biobank study to identify dementia and cardiometabolic condition cases and exclusion diagnoses

Figure S1. Study flow chart

Figure S2. Distribution of polygenic risk score for Alzheimer's Disease

Table S2. Association between cardiometabolic multimorbidity index and polygenic risk score

Figure S3. Risk of incident dementia based on cardiometabolic multimorbidity index and polygenic risk group.

Figure S4. Risk of incident dementia stratified by individual cardiovascular and metabolic conditions

Figure S5. Risk of incident dementia by cardiometabolic condition groups with myocardial infarction, atrial fibrillation and heart failure represented by a single heart disease variable

Table S3. Dementia risk by cardiometabolic index and polygenic risk quintiles

Table S4. Risk of incident dementia according to cardiometabolic multimorbidity and genetic risk, controlling for APOE Ɛ4 status

Figure S6. Risk of incident dementia by according to cardiometabolic multimorbidity index and genetic risk (additionally controlled for upstream risk factors)

Table S5. Dementia risk by cardiometabolic condition status with different follow-up periods

Figure S7. Risk of incident dementia by cardiometabolic disease status at baseline (unadjusted model)

Figure S8. Risk of incident dementia by according to cardiometabolic multimorbidity index and genetic risk (unadjusted model)

Table S6. Post-hoc comparisons examining the differences between individual indices of cardiometabolic multimorbidity and polygenic risk groups

Table S7. Associations between total hippocampal, total grey matter, total white matter hyperintensity volume with cardiometabolic multimorbidity index, polygenic risk and other baseline characteristics.

Table S1. Codes used in the UK Biobank study to identify dementia and cardiometabolic condition cases and exclusion diagnoses

	Algorithmically-derived	Self-report (includes non-cancer and treatment self report)	Illness code: ICD-10	Illness code: ICD-9
**Dementia**	-	1263	AD: F00, F00.0, F00.1, F00.2, F00.9, G30, G30.0, G30.1, G30.8, G30.9 VaD: F01, F01.0, F01.1, F01.2, F01.3, F01.8, F01.9, I67.3 FTD: F02.0, G31.0, Other codes for all-cause dementia: A81.0, F02, F02.1, F02.2, F02.3, F02.4, F02.8, F03, F05.1, F10.6, G31.1, G31.8	AD: 331.0 VaD: 290.4 FTD: 331.1 Other codes for all-cause dementia: 290.2, 290.3, 291.2, 294.1, 331.2, 331.5
**Myocardial Infarction**	All-cause myocardial infarction: 42000	-	-	-
**Stroke**	All-cause stroke: 42006	-	-	-
**Diabetes**	-	2443, 6153, 6177,	E10, E100, E101, E102, E103, E104, E105, E106, E107, E108, E109 E11, E110, E111, E112, E113, E114, E115, E116, E117, E118, E119, E12, E120, E121, E122, E123, E124, E125, E126, E127, E128, E129, E13, E130, E131, E132, E133, E134, E135, E136, E137, E138, E139, E14, E140, E141, E142, E143, E144, E145, E146, E147, E148, E149,E15, E150, E151, E152, E153, E154, E155, E156, E157, E158, E159	'25000', '25001', '25009', '25010', '25011', '25019', '25029', '2503', '2504', '2505', '25099'
**Atrial Fibrillation**	-	1471, 1483	I48	4273
**Heart Failure**	-	-	I110 , I130, I132, I255, I420, I425, I428, I429, I500, I501, I509	4254, 4280, 4281, 4289
**Peripheral vascular disease**		1067, 1087		
**Infection of the central nervous system**	-	1244	-	-
**Encephalitis**	-	1246	-	-
**Meningitis**	-	1247	-	-
**Amyotrophic lateral sclerosis**	-	1259	-	-

**Multiple Sclerosis**	-	1261	-	-
**Head Injury**	-	1266	-	-
**Subdural Haematoma**	-	1083	-	-
**Subarachnoid Haemorrhage**	-	1086	-	-

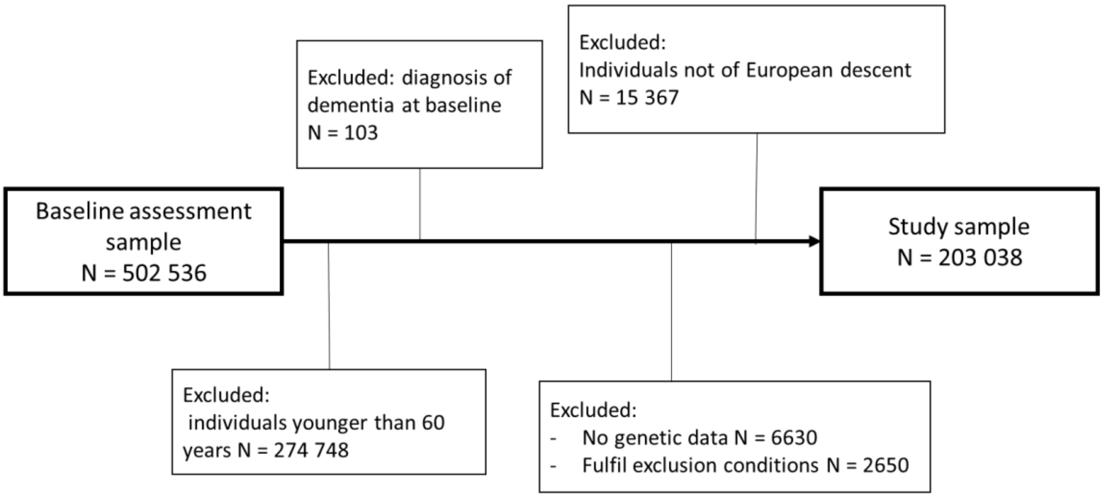

Figure S1. Study flow chart

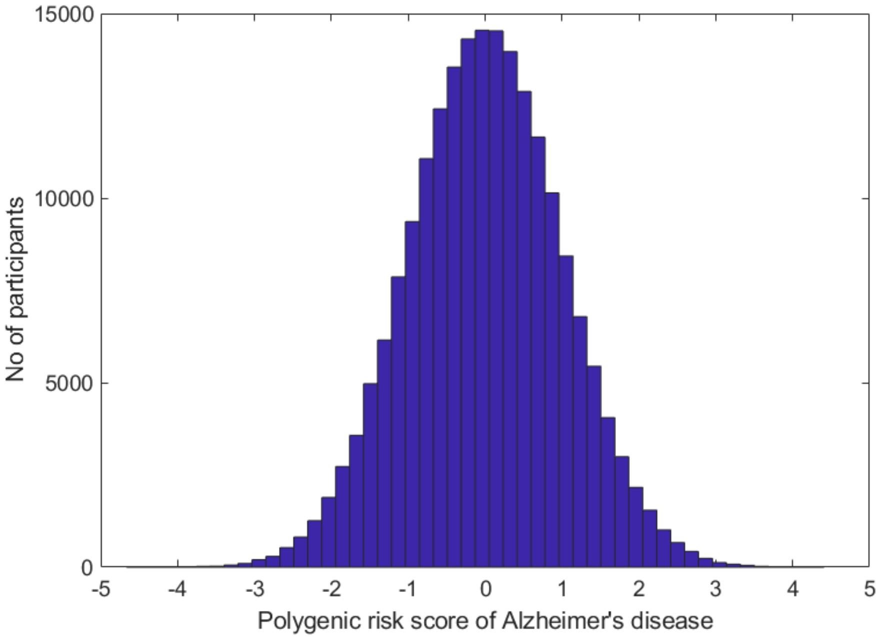

Figure S2. Distribution of polygenic risk score for Alzheimer's Disease.

Table S2. Association between cardiometabolic multimorbidity index and polygenic risk score

	**Polygenic risk score**	
	**β**	**p**
**CM index 1**	-5.1481e-07	0.103
**CM index 2**	7.0729e-07	0.459
**CM index 3**	-6.9069e-06	0.103

Note: β =unstandardised betas. Adjusted for age, sex, education, socioeconomic status, relatedness, number of alleles included in the polygenic risk score, first 20 principal components of ancestry and assessment centre.

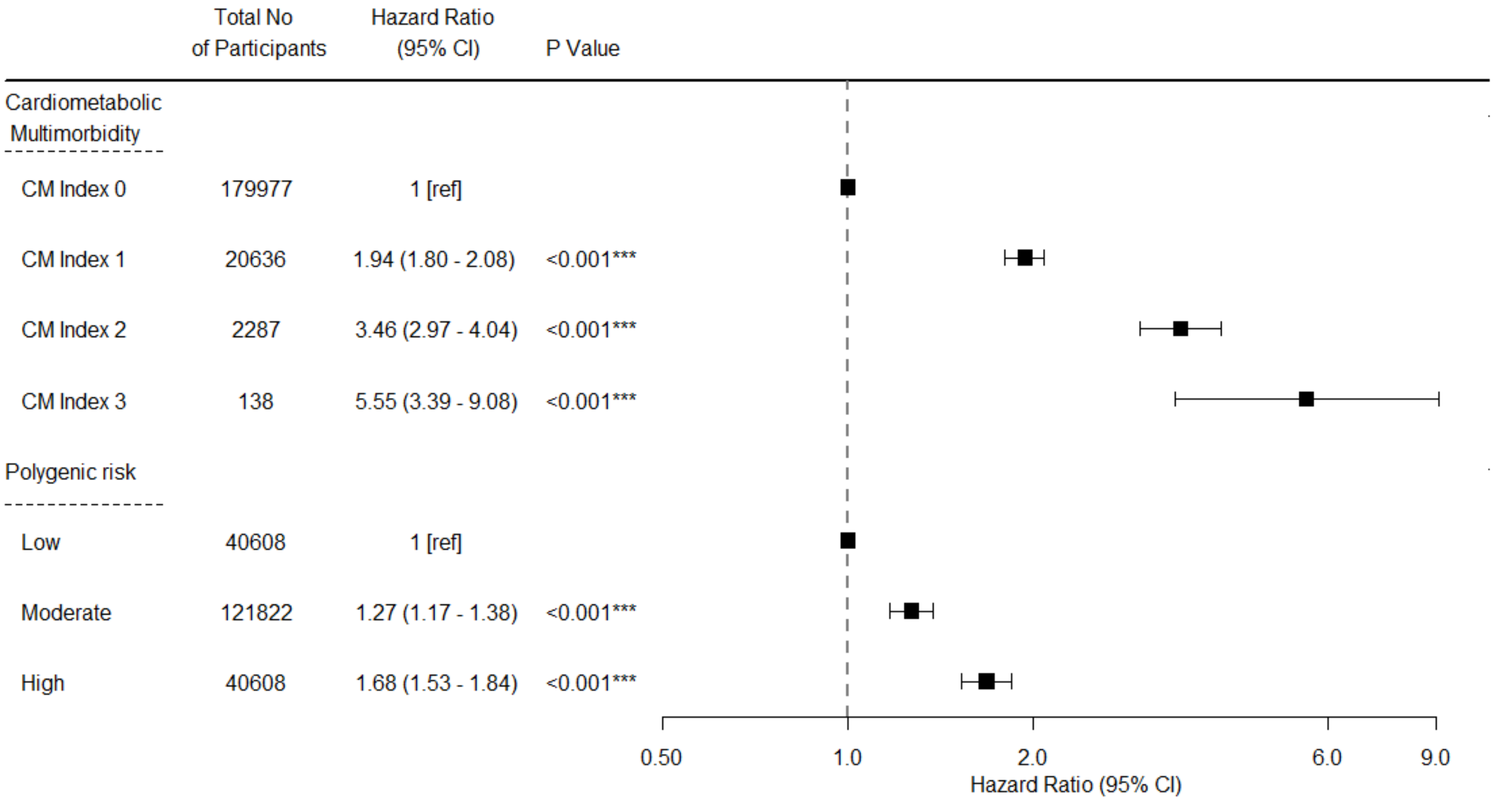

Figure S3. Risk of incident dementia based on cardiometabolic multimorbidity index and polygenic risk group.

Note: Adjusted for age, sex, education, socioeconomic status, relatedness, number of alleles included in the polygenic risk score, first 20 principal components of ancestry and assessment centre. Abbreviation: CM, cardiometabolic multimorbidity

	Total No of Participants	Hazard Ratio (95% CI)	P Value
Stroke	4247	2.05 (1.80 - 2.33)	<0.001***
Diabetes	13597	1.98 (1.82 - 2.15)	<0.001***
MI	7780	1.517 (1.36 - 1.69)	<0.001***
AF	7995	1.39 (1.12 - 1.71)	<0.001***
HF	2045	1.39 (1.13 - 1.71)	0.002**
PVD	632	1.39 (0.92 - 2.09)	0.117

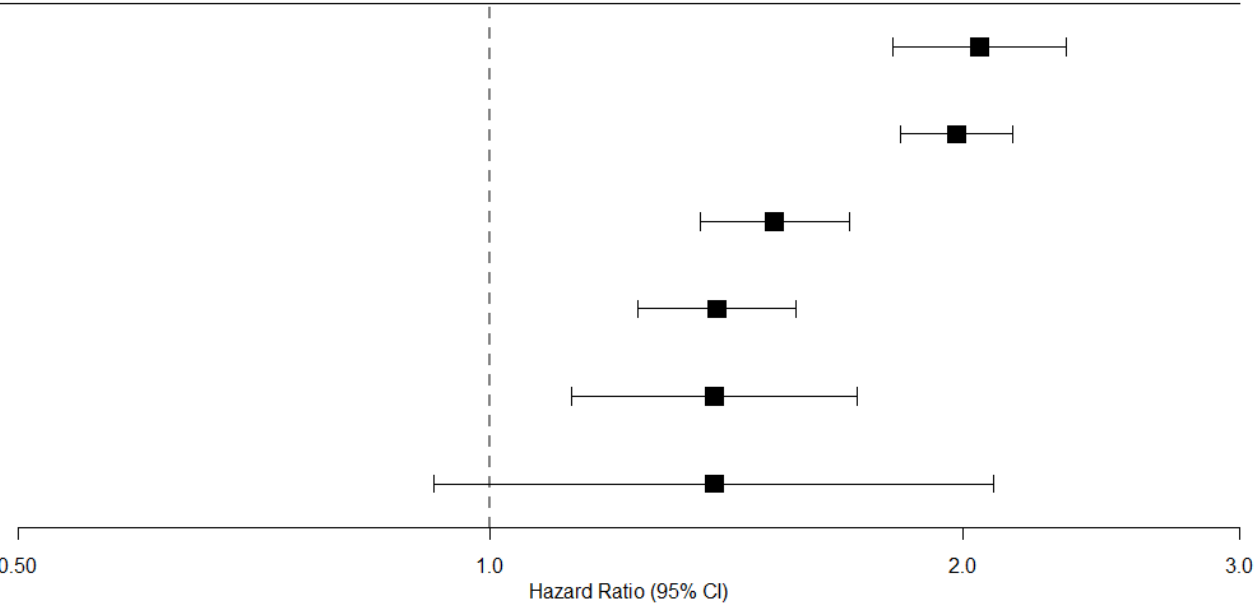

Figure S4. Risk of incident dementia stratified by individual cardiovascular and metabolic conditions

Note: Adjusted for age, sex, education, socioeconomic status, relatedness, number of alleles included in the polygenic risk score, first 20 principal components of ancestry and assessment centre. Abbreviation: MI, myocardial infarction; AF, atrial fibrillation; HF, heart failure; PVD, peripheral vascular disease.

	Total No of Participants	No. of dementia cases	Hazard Ratio (95% CI)	P Value
None	169313	3289	1 [ref]	
Heart Disease	16545	605	1.65 (1.51 - 1.81)	<0.001***
Diabetes	10289	436	1.98 (1.79 - 2.19)	<0.001***
Stroke	2717	127	2.09 (1.75- 2.19)	<0.001***
Heart Disease & Diabetes	2644	179	3.26 (2.80 - 3.80)	<0.001***
Stroke & Heart Disease	866	64	3.56 (2.62 - 4.30)	<0.001***
Stroke & Diabetes	419	42	4.63 (3.41 - 6.28)	<0.001***
Stroke & Heart Disease & Diabetes	245	24	5.04 (3.37 - 7.54)	<0.001***

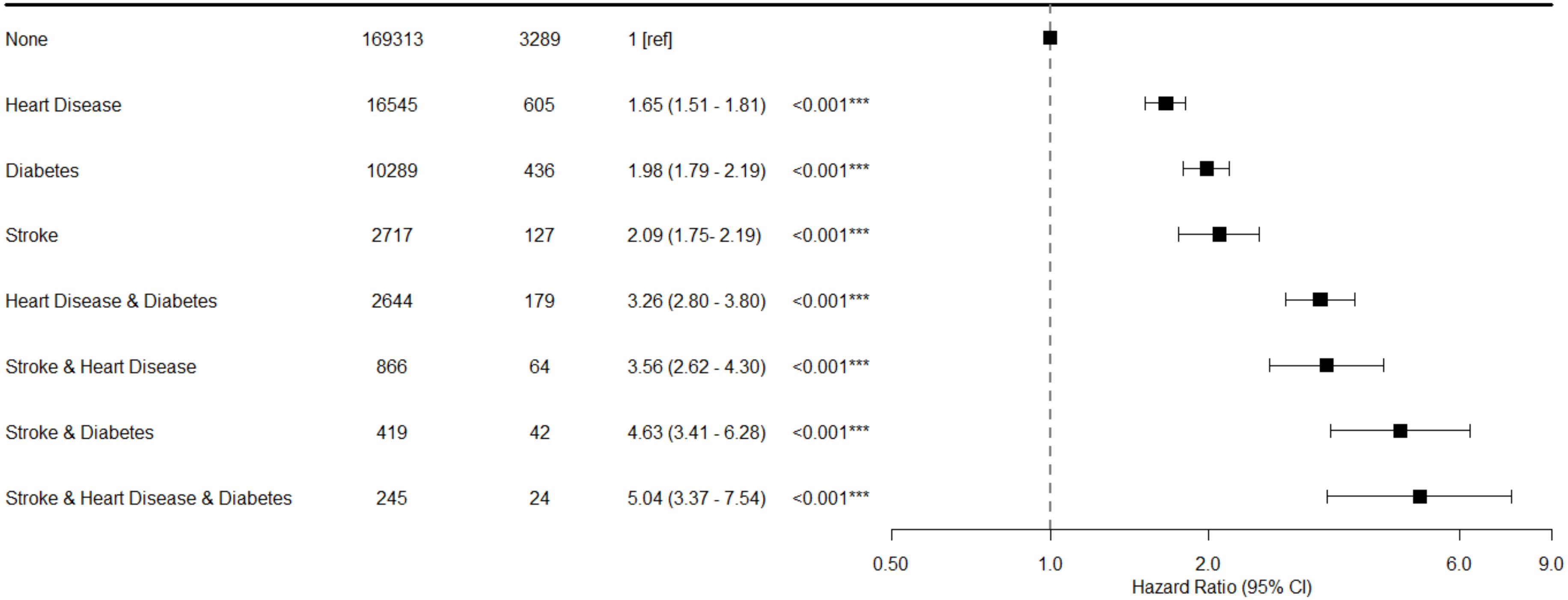

Figure S5. Risk of incident dementia by cardiometabolic condition groups with myocardial infarction, atrial fibrillation and heart failure represented by a single 'heart disease' variable

Note: Adjusted for age, sex, education, socioeconomic status, relatedness, number of alleles included in the polygenic risk score, first 20 principal components of ancestry and assessment centre.

Table S3. Dementia risk by cardiometabolic index and polygenic risk quintiles

	Dementia risk	
	**Hazard Ratio (95% CI)**	***P*-value**
CM Index 0	*1.00*	*[Ref]*
CM Index 1	1.94 (1.80 - 2.08)	***<0.001
CM Index 2	3.45 (2.96 - 4.03)	***<0.001
CM Index 3	1.94 (1.73 - 2.18)	***<0.001
PRS quint 1	*1.00*	*[Ref]*
PRS quint 2	1.20 (1.09 - 1.33)	***<0.001
PRS quint 3	1.22 (1.10 - 1.34)	***<0.001
PRS quint 4	1.38 (1.25 - 1.52)	***<0.001
PRS quint 5	1.68 (1.53 - 1.84)	***<0.001

Note: Adjusted for age, sex, education, socioeconomic status, relatedness, number of alleles included in the polygenic risk score, first 20 principal components of ancestry and assessment centre. Abbreviations: CM, cardiometabolic multimorbidity; PRS, polygenic risk score; CI, confidence interval

Table S4. Risk of incident dementia according to cardiometabolic multimorbidity and genetic risk, controlling for APOE Ɛ4 status

	Dementia risk	
	**Hazard Ratio (95% CI)**	***P*-value**
Low genetic risk		
CM Index 0	1.00	[Ref]
CM Index 1	1.98 (1.65 - 2.38)	***<0.001
CM Index 2+	3.55 (2.43 - 5.20)	*0.004
Moderate genetic risk		
CM Index 0	1.15 (1.04 - 1.26)	***<0.001
CM Index 1	2.33 (2.07 - 2.62)	***<0.001
CM Index 2+	4.16 (3.40 - 5.10)	***<0.001
Moderate genetic risk		
CM Index 0	1.42 (1.28 - 1.57)	***<0.001
CM Index 1	2.35 (2.00 - 2.76)	***<0.001
CM Index 2+	4.90 (3.63 - 6.61)	***<0.001

Note: Adjusted for age, sex, education, socioeconomic status, relatedness, number of alleles included in the polygenic risk score, first 20 principal components of ancestry and assessment centre. Abbreviations: CM, cardiometabolic multimorbidity; PRS, polygenic risk score; CI, confidence interval

Subgroup	Total No of Participants	No. of dementia cases	Hazard Ratio (95% CI)	P Value
Low genetic risk				
CM Index 0	36006	556	1 [ref]	
CM Index 1	4154	145	1.97 (1.61 - 2.40)	<0.001***
CM Index 2+	448	28	3.34 (2.20 - 5.05)	<0.001***
Moderate genetic risk				
CM Index 0	107933	2121	1.29 (1.16 - 1.43)	<0.001***
CM Index 1	12415	566	2.57 (2.25 - 2.95)	<0.001***
CM Index 2+	1474	116	4.35 (3.45 - 5.49)	<0.001***
High genetic risk				
CM Index 0	36038	982	1.77 (1.58 - 1.98)	<0.001***
CM Index 1	4067	205	2.64 (2.19 - 3.17)	<0.001***
CM Index 2+	503	47	5.37 (3.85 - 7.50)	<0.001***

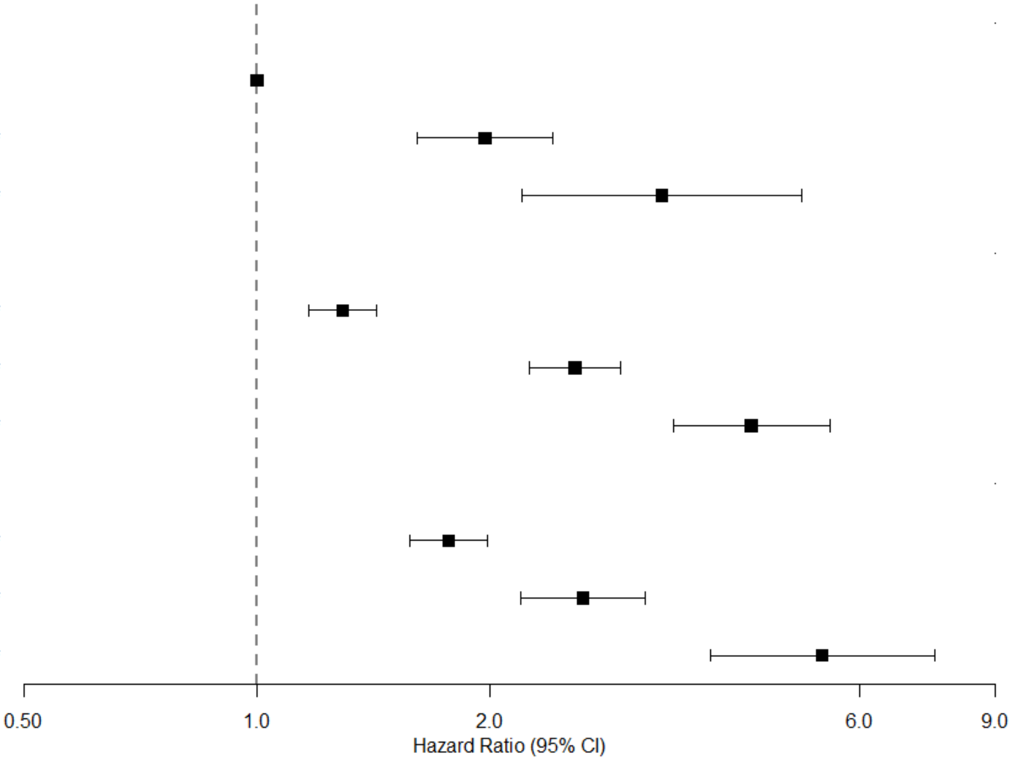

Figure S6. Risk of incident dementia by according to cardiometabolic multimorbidity index and genetic risk (additionally controlled for upstream risk factors)

Shown are the hazard ratios for incident dementia, according to cardiometabolic multimorbidity index and genetic risk with further adjustment for markers of upstream cardiovascular and metabolic risk factors (systolic blood pressure, total cholesterol, body mass index and glycosylated haemoglobin levels) in addition to for age, sex, education, socioeconomic status, relatedness, number of alleles included in the polygenic risk score, first 20 principal components of ancestry and assessment centre. *Abbreviations: CM, cardiometabolic multimorbidity*

Table S5. Dementia risk by cardiometabolic condition status with different follow-up periods

Subgroup	Dementia risk at 10 years			Dementia risk between 10 - 14 years		
	**No. of dementia cases**	**Hazard Ratio (95% CI)**	***P*-value**	**No. of dementia cases**	**Hazard Ratio (95% CI)**	***P*-value**
No cardiometabolic conditions (baseline)	*2308*	*1.00*	*[Ref]*	*1352*	*1.00*	*[Ref]*
Stroke	112	2.45 (2.05 - 2.94)	***<0.001	45	1.87 (1.41 - 2.48)	***<0.001
MI	164	1.79 (1.53 - 2.09)	***<0.001	71	1.56 (1.23 - 1.97)	***<0.001
Diabetes	329	1.94 (1.73 - 2.18)	***<0.001	195	2.29 (1.97 - 2.65)	***<0.001
Stroke & MI	36	3.41 (2.28 - 5.10)	***<0.001	14	4.37 (2.53 - 7.55)	***<0.001
Stoke & Diabetes	51	4.70 (3.39 - 6.50)	***<0.001	15	4.09 (2.46 - 6.81)	***<0.001
MI & Diabetes	74	3.02 (2.34 - 3.90)	***<0.001	33	3.69 (2.60 - 5.24)	***<0.001
Stroke & MI & Diabetes	15	7.34 (4.41 - 12.21)	***<0.001	1	1.28 (0.18 - 9.12)	0.80

Note: Adjusted for age, sex, education, socioeconomic status, relatedness, number of alleles included in the polygenic risk score, first 20 principal components of ancestry and assessment centre. Abbreviations: MI, myocardial infarction; CI, confidence interval

	Total No of Participants	No. of dementia cases	Hazard Ratio (95% CI)	P Value
None	179977	3659	1 [ref]	
MI	5881	235	2.13 (1.87 - 2.43)	<0.001***
Diabetes	11592	524	2.39 (2.18 - 2.62)	<0.001***
Stroke	3163	157	2.64 (2.25- 3.10)	<0.001***
MI & Diabetes	1341	91	4.20 (3.41 - 5.17)	<0.001***
Stroke & MI	420	34	4.85 (3.46 - 6.81)	<0.001***
Stroke & Diabetes	526	50	5.71 (4.32 - 7.55)	<0.001***
Stroke & MI & Diabetes	138	16	8.40 (5.14 - 13.72)	<0.001***

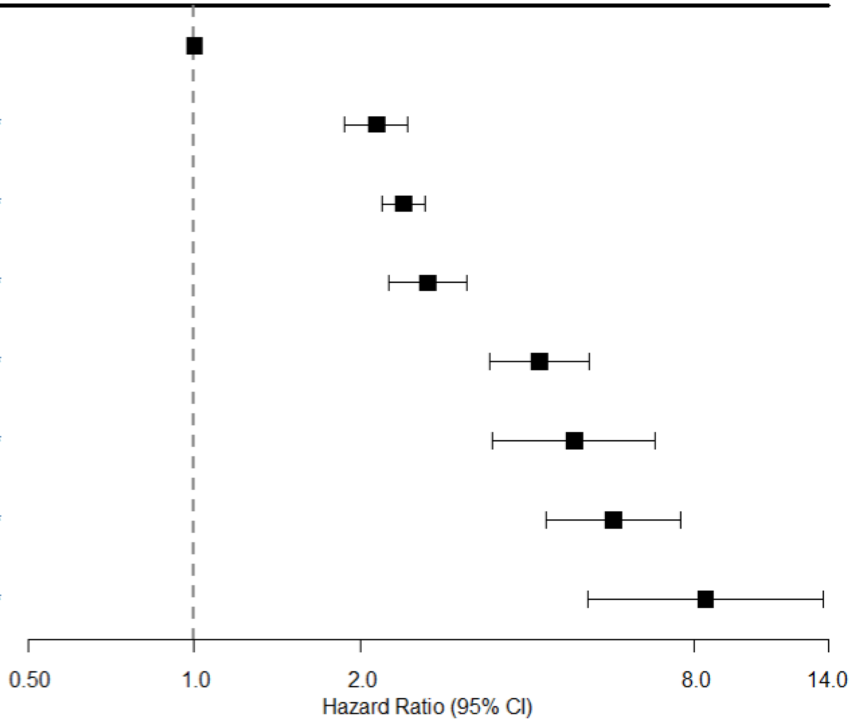

Figure S7. Risk of incident dementia by cardiometabolic disease status at baseline (unadjusted model)

Shown are hazard ratios for incident dementia, associated with mutually exclusive groupings of cardiometabolic conditions. The model was unadjusted.
Abbreviations: MI, myocardial infarction

Subgroup	Total No of Participants	No. of dementia cases	Hazard Ratio (95% CI)	P Value
Low genetic risk				
CM Index 0	36006	556	1 [ref]	
CM Index 1	4154	145	2.42 (2.02 - 2.90)	<0.001***
CM Index 2+	448	28	4.97 (3.40 - 7.26)	<0.001***
Moderate genetic risk				
CM Index 0	107933	2121	1.27 (1.16- 1.40)	<0.001***
CM Index 1	12415	566	3.19 (2.84 - 3.59)	<0.001***
CM Index 2+	1474	116	6.34 (5.19 - 7.75)	<0.001***
High genetic risk				
CM Index 0	36038	982	1.77 (1.59 - 1.96)	<0.001***
CM Index 1	4067	205	3.54 (3.02 - 4.16)	<0.001***
CM Index 2+	503	47	7.93 (5.89 - 10.68)	<0.001***

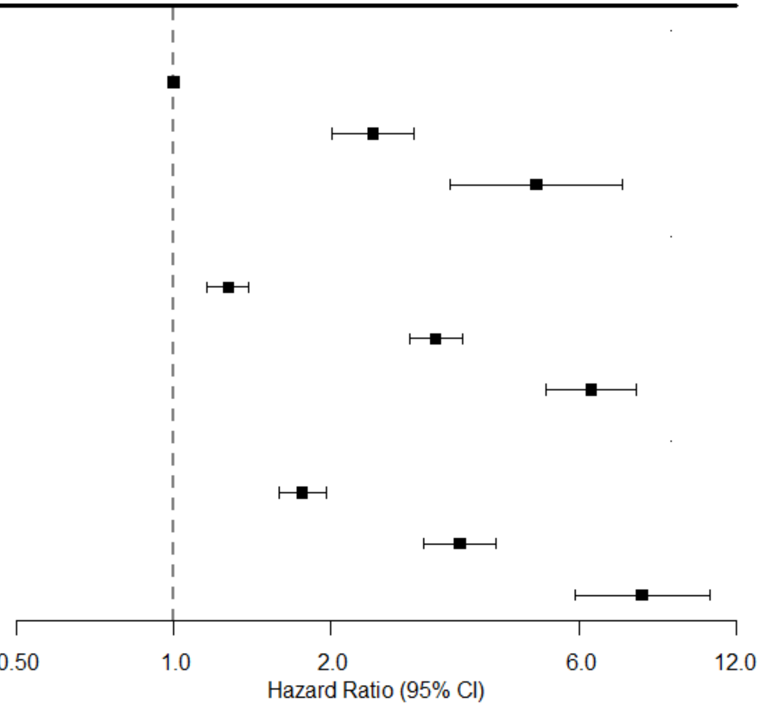

Figure S8. Risk of incident dementia by according to cardiometabolic multimorbidity index and genetic risk (unadjusted model)

Shown are the hazard ratios for incident dementia, according to cardiometabolic multimorbidity index and genetic risk. The reference group were participants with low genetic risk and a CM Index of zero. Horizontal bars indicate 95% confidence intervals. This model was unadjusted. *Abbreviations: CM, cardiometabolic multimorbidity*

Table S6. Post-hoc comparisons examining the differences between individual indices of cardiometabolic multimorbidity and polygenic risk groups

	Total Hippocampal Volume			Total Grey Matter Volume			Total White Matter Hyperintensity Volume		
	**F**	**p**		**F**	**p**		**F**	**p**	
**CM Index**	10.70	*< .001		55.65	*< .001		4.48	*0.011	
**Polygenic Risk**	3.45	*0.032		1.67	0.189		0.98	0.376	
**CM index * Polygenic Risk**	1.59	0.174		0.87	0.334		0.42	0.795	
**Post-hoc analysis**	**t**	**p_{holm}**	**p_{tukey}**	**t**	**p_{holm}**	**p_{tukey}**	**t**	**p_{holm}**	**p_{tukey}**
**CM Index**									
CM0 – CM1	3.73	*< .001	*< .001	10.54	*< .001	*< .001	-2.76	*0.017	*0.016
CM0 – CM2+	2.79	*0.016	*0.015	3.093	*0.006	*0.006	-1.20	0.691	0.453
CM1 – CM2+	1.826	0.204	0.161	0.517	0.990	0.821	-0.56	0.990	0.841
**Polygenic risk**									
Low – mod	2.00	0.138	0.113	1.656	0.217	0.171	-0.84	0.990	0.678
Low – high	2.48	*0.039	*0.035	0.919	0.705	0.461	-1.39	0.493	0.346
Mod – high	1.030	0.909	0.558	-0.408	0.990	0.971	-0.82	0.990	0.688

Post-hoc analysis of relationship between individual indices of CM index and polygenic risk groups assessed with Holm–Bonferroni correction applied to account for multiple comparisons and with Tukey pairwise analysis.

Abbreviations: CM, cardiometabolic multimorbidity; mod, moderate

Table S7. Associations between total hippocampal, total grey matter, total white matter hyperintensity volume with cardiometabolic multimorbidity index, polygenic risk and other baseline characteristics.

	Total Hippocampal Volume		Total Grey Matter Volume		Total White Matter Hyperintensity Volume	
	β	p	β	p	β	p
**CM Index**	-102.977	*< .001	-12511.345	*< .001	0.100	*< .001
**Polygenic Risk**	-23.069	*0.041	-785.262	0.131	0.004	0.629
Sex	-20.202	0.171	-2356.547	< .001	-0.125	< .001
Education						
Upper Secondary	-19.031	0.450	-308.849	0.787	-0.018	0.385
Vocational	-3.723	0.901	-79.918	0.953	0.018	0.451
Higher ed	16.744	0.341	1175.365	0.142	-0.047	< .001
Socio						
Quintile 2-4	1.011	0.953	-1071.334	0.171	0.003	0.817
Quintile 5	-35.958	0.149	-3317.742	0.003	0.044	0.031
Relatedness	6.549	0.676	1327.823	0.062	0.005	0.677
Allele number	0.003	0.850	0.808	0.208	0.000	0.383
pc1	-3.304	0.396	438.139	0.013	0.001	0.871
pc2	-3.580	0.342	155.665	0.363	0.002	0.430
pc3	-0.446	0.914	-32.310	0.864	-0.001	0.843
pc4	-0.365	0.877	228.648	0.032	0.002	0.379
pc5	-1.294	0.289	-68.036	0.220	0.002	0.117
pc6	1.306	0.755	101.619	0.593	-0.003	0.407
pc7	0.726	0.813	87.500	0.531	0.003	0.260
pc8	1.607	0.633	-132.038	0.387	0.004	0.139
pc9	-1.498	0.431	-17.008	0.844	0.002	0.316
pc10	0.782	0.837	114.314	0.507	0.001	0.656
pc11	0.280	0.922	15.310	0.906	0.004	0.076
pc12	1.237	0.746	38.719	0.823	0.003	0.327
pc13	2.990	0.505	-211.409	0.299	-0.005	0.130
pc14	7.602	0.002	241.725	0.026	0.000	0.890
pc15	5.194	0.210	489.477	0.009	-0.003	0.299
pc16	-5.251	0.035	-207.580	0.067	0.000	0.819
pc17	-4.732	0.196	82.284	0.620	-0.001	0.658
pc18	4.776	0.048	150.318	0.170	-0.004	0.033
pc19	-5.994	0.016	-331.785	0.003	-0.002	0.390
pc20	0.957	0.706	-13.321	0.908	0.000	0.812

Note: β = unstandardised betas coefficients. All volume measures have been deconfounded for age and age^2 at imaging visit, a scaling factor for head size and imaging site. White matter hyperintensity volume was log-transformed due to a skew distribution.

Abbreviations: CM, cardiometabolic multimorbidity; pc, principal component
